# Advancing experimental fish welfare: insights from the Swiss fish welfare meeting 2026

**DOI:** 10.3389/fphys.2026.1917263

**Published:** 2026-10-28

**Authors:** Bettina Kirchmaier, Livia D’Angelo, Alexis Buatois, Gary Delalay, Nicolas Diserens, Rainer W. Friedrich, Britta Lundström-Stadelmann, Joana Monteiro, Yannick Rohrer, Linda Tschirren, Bernhard Voelkl, Guillaume Valentin, Andrina Zbinden-Hauzenberger, Sharon Wismer, Irene Adrian-Kalchhauser

**Affiliations:** 1 Goethe-Universitat Frankfurt am Main, Frankfurt, Germany; 2 Universita degli Studi di Napoli Federico II, Naples, Italy; 3 Eawag, Dübendorf, Switzerland; 4 Universitat Bern, Bern, Switzerland; 5 Friedrich Miescher Institute for Biomedical Research and University of Basel, Basel, Switzerland; 6 Fundacao Champalimaud, Lisbon, Portugal; 7 Fair-Fish, Zürich, Switzerland; 8 Nofima AS, Tromsø, Norway; 9 Ecole Polytechnique Federale de Lausanne, Lausanne, Switzerland; 10 Universite de Fribourg, Fribourg, Switzerland

**Keywords:** aquaculture, culture of care, cumulative assessment, environmental enrichment, euthanasia, knowledge exchange, refinement, regulatory frameworks

## Abstract

Fish are widely used in research and aquaculture, yet many aspects of their welfare remain poorly understood. Recent years have seen a shift from health and disease prevention as cornerstones of wellbeing toward concepts of welfare that include behavior, affective states, cumulative lifetime experiences, and positive welfare. Current challenges relate to the taxonomic diversity of fishes, the dominance of zebrafish as model species, and the translation of welfare concepts across experimental, applied, and industrial settings. Knowledge gaps remain regarding welfare assessment, environmental enrichment, euthanasia, pain management, and the implementation of evidence-based refinement measures. Here we summarize the outcomes of the Swiss Fish Welfare Symposium 2026, which brought together researchers, caretakers, veterinarians, regulators, and industry. Participants identified drivers of change such as a Culture of Care, improved knowledge exchange, and incentive structures to reward refinement. Presentations emphasized the need for welfare research under realistic husbandry conditions, the development of cumulative and species-specific welfare assessment tools, and the generation of evidence for housing, enrichment, analgesia, euthanasia, and rehoming practices. Speakers stressed that recommendations developed under idealized conditions often fail to reflect the practical realities of research facilities and aquaculture systems. The symposium revealed a research field in transition from compliance-based approaches toward a vision of welfare that encompasses both animal and human well-being. Participants identified collaboration, systematic use of existing welfare data, and research beyond zebrafish as critical priorities. More broadly, discussions highlighted the need to bridge gaps between fundamental research, regulation, husbandry practice, and industry to achieve evidence-based and implementable improvements in fish welfare.

## 1 Introduction

The meeting on June 8^th^ 2026 at the EPFL Lausanne brought together ~50 participants from 5 European countries, 9 Swiss research institutions, 4 international research institutions, 3 Swiss fish linked institutions, 1 company, and 3 administrative institutions. About half of the attendees identified as fish care takers or fish facility managers, which illustrates the need for continued education events for this profession. The figure logo was kindly provided by Pia Cigler (**Figure 1**).

**Figure 1:**
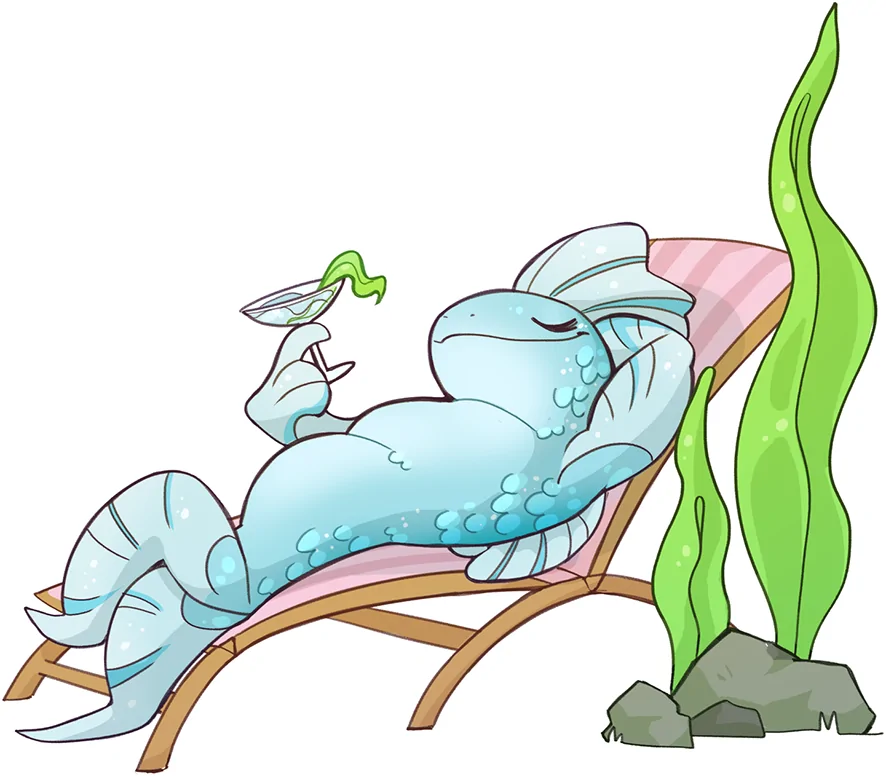
Logo of the meeting. Original artwork, (c) Pia Cigler.

## 2 The human side of fish welfare

The first session, opened by Britta Lundström-Stadelmann from the Institute for Parasitology in Bern, was dedicated to various aspects of a Culture of Care. Culture of Care represents a commitment to improving animal welfare, scientific quality, staff well-being, and transparency. Within the fish research community, topics relating to Culture of Care, such as ethics and compassion fatigue, are currently not widely discussed.

Factors hindering the development of a Culture of Care (CoC) in any species include rigid hierarchies, poor communication, high workloads, time and career pressures, and an excessive focus on procedures and compliance (Ferrara et al., 2022). Importantly, the CoC concept extends beyond animal welfare and refinement measures, and beyond regulatory compliance. Elements also include transparency, open communication across all roles, mutual respect, care for both animals and staff, inclusion, and scientific integrity (Abelson et al., 2023). A successful CoC relies on a safe environment built on trust, where all team members feel able to discuss the emotional and psychological aspects of their work (Morahan et al., 2024). Such conversations can be challenging or unusual in research settings but are essential for fostering mutual understanding and support. When established successfully, these elements of a CoC can also become a powerful driver of team cohesion.

In addition, embedding a CoC into everyday routines improves an institution’s ability to respond to unexpected or particularly challenging situations, exemplified by euthanasia directives during the COVID-19 pandemic. CoC is also linked to scientific quality: reducing stress in animals reduces stress in experimenters and thus indirectly contributes to more reliable, less variable data (Bailey, 2018).

Practical measures can reinforce these values. Examples presented included recognizing the often demanding and emotionally challenging work of animal caretakers through initiatives such as animal caretaker appreciation days, involving care staff in project planning, conducting experiment debriefings, or holding memorials that acknowledge the contribution of research animals. However, Lundström-Stadelmann emphasized that a CoC cannot be reduced to a series of events. It must be embedded in daily practice and maintained through continuous investment and commitment.

Importantly, lessons learned in species with a more established CoC, such as laboratory rodents, provide a useful basis for developing CoC approaches in fish research. Regardless of species, shared responsibility, recognition of emotional burden, a culture that encourages speaking up, appreciation of the observations and expertise of animal caretakers, and open communication across roles all strengthen both animal welfare and scientific quality. Likewise, many of the obstacles to a Culture of Care—hesitation, silence, workload pressures, hierarchical structures, and the dominance of quantitative performance metrics—are not species-specific but are deeply rooted in academic culture itself (Ameli and Krämer, 2024; Aubert Bonn et al., 2022; Williams, 2023).

## 3 Strengthening fish welfare networks

The Culture of Care concept encompasses the principles of Replacement, Reduction, and Refinement,but extends beyond into everyday practice: it involves all staff in decision-making processes, promotes continuous reflection on animal welfare, and relies on the active sharing of knowledge and experience between institutions. It is fundamentally an attitude and a commitment that goes beyond legal requirements (Brown et al., 2018). In Switzerland, the Swiss Culture of Care Network provides a platform for education, exchange, and collaboration (Swiss Culture of Care Network, n.d.). As Andrina Zbinden-Hauzenberger, animal welfare officer at the University of Fribourg, explained, the network is currently expanding its activities to better address fish welfare, and to reflect the growing use of fish in research according to national statistics. This is not trivial because indicators of well-being, distress, and pain are more difficult to recognize and interpret in fish than in mammals (Sneddon and Roques, 2023; Sneddon, 2009), and the perception and presentation of fish as the replacement model leads to an underappreciation of the specific needs of the species group. To facilitate the sharing of expertise, the Swiss Culture of Care Network has established a zebrafish refinement group that aims to promote information exchange, harmonization of protocols, and the development of recommendations.

Three dedicated working groups currently focus on (I) anesthesia, analgesia and euthanasia; (II) housing and enrichment; and (III) genetically modified animal monitoring and documentation. All three working groups have identified a lack of scientific evidence as obstacles for their respective areas. For example, methods of analgesia or euthanasia that are commonly used in research are only conditionally permitted by law — not because they are ineffective, but because the supporting research data are insufficient (Sneddon et al., 2024). For the same reason, zebrafish are currently not included in many national pain-management guidelines for experimental animals, even though they have been lined out by FELASA (Sneddon et al., 2024). Similar issues occur with genetically modified animals, housing, and environmental enrichment. In Switzerland, the Animal Protection Ordinance does not prescribe zebrafish-specific husbandry parameters because of the sparse scientific evidence (Swiss Federal Council, 2008). Participants considered this limited species-specific guidance to reflect, at least partly, gaps in the underlying scientific evidence. The network therefore aims to exchanging best practices, but also to address these knowledge gaps.

## 4 Creating incentives for welfare

How can we promote the establishment of a genuine culture of care and the adoption of refinement measures? Bernhard Voelkl from the Veterinary Public Health Institute in Bern pointed out that the system currently relies largely on “push” mechanisms: laws, regulations, and standards that define the minimum requirements for animal welfare. While these measures effectively ensure compliance, they provide little incentive to go beyond the minimum.

The CARE Awards, which will be awarded starting 2027, represent a novel and complementary “pull” approach with a specific focus on publications. Developed in response to identified barriers to change, such as costs, established habits, institutional inertia, and existing reward structures, the awards are designed to increase the visibility and recognition of refinement efforts. They thus award welfare improvements only in combination with dissemination. Key domains for refinement that are relevant for the CARE Awards are husbandry, experimental procedures, and human–animal interactions. To qualify for an award, improvements in one or more of these areas must be clearly reported in the methods section or supplementary material of a scientific publication. Bronze, Silver, and Gold awards will acknowledge increasing levels of reported refinement. Importantly, eligibility requires the documented implementation of a refinement method in research – method developments are not awarded.

## 5 Rehoming as refinement

Refinement and welfare extend beyond the completion of experiments. Based on the FELASA recommendation for rehoming (Ecuer et al., 2023), European legislation provides for the rehoming of experimental animals as an alternative to euthanasia (European Parliament and Council of the European Union, 2010). Bettina Kirchmaier from the Goethe University in Frankfurt described their practical experience from an ongoing zebrafish rehoming programme in which wild-type fish are provided to external caretakers, including schools and kindergartens. She explained how implementing rehoming programs comes with several practical and regulatory challenges. For example, in the programme presented, genetically modified animals were not eligible for external rehoming. She recommends to maintain such animals in large ornamental aquaria, where they can serve as stock for educational purposes or are allowed to live out their natural lifespan.

For fish that can be rehomed to external caretakers, education and support for new fish owners are key. Rehoming to inexperienced private individuals requires appropriate guidance, which is complicated by a lack of practical, accessible information for first-time zebrafish keepers.

Schools and kindergartens represent a particularly interested target group. Keeping animals in educational settings has been associated with benefits for social functioning and engagement, including in neurodiverse students (O'Haire et al., 2013, 2014). In the presented example, collaboration with bioscience educators in training proved particularly fruitful. Student teachers developed educational materials and teaching modules covering phenotypes, behavior, water chemistry, and the nitrogen cycle. The fish also provided opportunities for simple observational studies of pupils, such as odor-mediated behaviors.

Importantly, welfare considerations remained central to the program. Participants can return both fish and equipment at any timepoint if they are no longer able to provide appropriate care (e.g. they no longer have the space or time for fish and tank maintenance) to ensure that animal welfare standards are maintained throughout the animals’ lives. According to the experience presented, the programme currently operates at capacity and receives more requests than it can accommodate.

## 6 Regulatory developments

The implementation of welfare advances ultimately depends on legal and regulatory support. Livia D’Angelo from the University of Naples outlined recent clarifications relating to zebrafish contained in an amendment to Directive 2010/63/EU on the protection of animals used for scientific purposes (European Commission, 2024). The amendment, whose provisions apply from 4 December 2026, introduces specific water-quality and lighting requirements for zebrafish and stipulates that water volumes smaller than 1 L shall not be used for adult zebrafish and that stocking densities shall not exceed 10 adult fish/L. Depending on how these provisions are interpreted and implemented by EU member states, they may have practical implications for breeding systems such as mating slope grids, which reduce the available swimming space below 1 liter. Another area covered by the amendment is killing methods. The list of permitted approaches has been broadened and, depending on species and fish size, the amended Annex IV permits methods including anesthetic overdose, concussion/percussive blow to the head, electrical stunning and, specifically for zebrafish under defined conditions, hypothermic shock (European Commission, 2024). The latter is permitted under specified conditions based on the lack of cold nociceptors in salmonids and antifreeze gene expression in adults, and the absence of recovery after five minutes (Köhler et al., 2017; Wallace et al., 2018). For hypothermic shock, the amendment specifies zebrafish ≥16 dpf and ≤5 cm body length, a temperature ≤4 °C, a temperature difference from holding conditions of ≥20 °C, no direct contact with ice, and a minimum exposure time of 5 min. The amendment further requires killing to be completed by confirmation of permanent cessation of circulation, destruction of the brain, neck dislocation, exsanguination, or confirmation of rigor mortis, using a method appropriate for the species. This step is relevant given the persistence of certain reflexes following approved killing methods, and doubts regarding a reliable differentiation between death and paralysis (Mocho et al., 2025).

In parallel to the amendment, the FELASA recommendations for laboratory fish state that euthanasia methods should be species-appropriate, rapid, minimally stressful, compatible with research objectives, easy to learn, and robust against user error.

For zebrafish, loss of opercular movement, righting and startle reflexes can be used in assessing progression towards euthanasia, although disappearance of individual reflexes alone may not reliably demonstrate death (Mocho et al., 2025).

## 7 Species differences in euthanasia

A major challenge when developing recommendations for fish euthanasia beyond zebrafish, which includes pet fish, is the lack of reliable data. In contexts such as fisheries and ornamental pet fish, clove oil is widely used and has been recommended for anaesthesia and euthanasia (Javahery et al., 2012; Priborsky and Velisek, 2018). Nicolas Diserens from the Institute for Fish and Wildlife Health in Bern presented preliminary, unpublished results comparing clove oil and tricaine (MS-222) in four ornamental fish species. According to the presenter, early observations point to species-specific differences in response, and they cautioned that the suitability of clove oil for euthanasia should not be assumed to generalise across ornamental fish species.

Finding an appropriate, suited euthanasia method for aquatic organisms presents a challenge also for the food industry. Killing decapod crustaceans by immersing them alive in boiling water raises substantial animal-welfare concerns and, in Switzerland, is prohibited without prior stunning (Swiss Federal Council, 2008). Gary Delalay from the Institute for Fish and Wildlife Health in Bern presented preliminary findings from an ongoing literature review assessing the indicators used to determine loss of consciousness in crustaceans and their reliability.

## 8 Innovation for welfare

Technological innovation carries great potential to improve welfare for experimental fish. A practical example of an innovation for welfare was presented by Guillaume Valentin from the EPFL Lausanne. Valentin presented preliminary, unpublished data indicating that egg retention was not associated with female age in the population examined. Instead, the condition occurred particularly frequently in routine breeding stock tanks and appeared to be associated with prolonged intervals between breeding events.

Their team therefore developed and patented an *in-situ* breeding solution for the widespread tecniplast tank setup which saves time and allows animals to stay in their husbandry tank. In essence, a sloped chamber is placed into the tank overnight and allows the recovery of eggs in the morning, without removing the tank from the rack. Simple modifications also allow the collection of synchronized embryos. For example, zebrafish can be kept beneath the slope overnight, and are allowed to use the slope in the morning. Currently, enrichment structures of similar design principles are being tested to promote habituation to the slopes and further improve the acceptance of the system. Preliminary observations with the *in-situ* breeding system suggest that it reduces the occurrence of egg-bound individuals while allowing animals to remain in their husbandry tank. Current prototypes are produced using 3D printing, but commercial versions are expected to become available soon.

## 9 From lab to industry

Much of the fundamental research on fish welfare focuses on zebrafish and small experimental scales. This does not reflect the enormous taxonomic breadth of fishes used in research, and also does not account for the scales of aquaculture industries. Species such as trout, medaka, stickleback, zebrafish or salmon are evolutionarily more distant from one another than mice are from humans (Kumar et al., 2022), while commonly used group sizes range from a few individuals in research to tens or hundreds of thousands in industry. How can welfare concepts be translated across such scales?

Linda Tschirren from the Norwegian research institute Nofima discussed the challenges of translating welfare principles across fundamental research, applied research, and industry. These sectors currently define welfare differently. Fundamental animal welfare science operates within the framework of the five domains (Health, Nutrition, Environment, Behavior, and Mental State) and is currently expanding toward positive animal welfare concepts (Fife-Cook and Franks, 2019; Mellor et al., 2020). The identification of high-and low-arousal states as well as positive and negative valence allows the description of an individual’s mental states as fear, excitement, relaxation, or boredom. Aquaculture currently focuses strongly on functional outcomes and equates welfare to the absence of disease or injury; welfare serves as quality assurance measure, but welfare-related data – that are routinely collected to satisfy regulatory requirements - are not analyzed. Unsurprisingly, economic considerations are central: welfare interventions must either provide a measurable return on investment or at least be cost-neutral. Building bridges between research and industry, connecting their fundamentally different approaches and translating between these sectors remains challenging. Biological indicators that are informative under laboratory conditions may not be relevant in high-density production environments, may be difficult to scale, or may not be sufficiently robust or cost-effective for routine use. Even the interpretation and consequences of welfare indicators may differ between contexts, as is evident for cortisol data. In research, low cortisol concentrations indicate the absence of stress. While this does not necessarily imply positive welfare, low cortisol is often used as evidence of the absence of poor welfare (Ellis et al., 2012). Inversely, in an industrial setting, a certain level of stress early in life may promote adaptive coping mechanisms and improve resilience later in life, and constantly low cortisol levels may therefore not be considered inherently beneficial. The industry’s current focus is to move from a narrow focus on health toward a broader understanding of welfare, and from single-point assessments toward cumulative welfare evaluation across the lifetime of the fish.

## 10 Monitoring welfare

A tool to monitor the effects of environment on fish welfare continuously, and potentially in a cumulative manner, are score sheets (Bugnon et al., 2016; Thallmair et al., 2026). Bettina Kirchmaier from the Goethe University in Frankfurt presented preliminary results of a life-long assessment of ageing zebrafish populations using a recent score sheet specifically designed to accommodate fish and their diversity (Flueck-Giraud et al., 2025). The assessment included several viable knockout lines and reporter lines that were previously considered free from morphological impairments, as well as the wildtype lines AB and TL. Age-synchronized adults were scored monthly between 6 and 24 months of age. Common ageing signs in the wildtype lines AB and TL included emaciation and dorsal curvature, but also defects on the skin (AB) and fins (TL). In this dataset, first signs of ageing became apparent from 12 months onwards, including mortalities, and increased in prevalence by 18 months. These data are consistent with the FELASA–AALAS recommendations for monitoring and reporting laboratory fish health status, which recommend that zebrafish should not be maintained in the main recirculating system beyond 18 months (Mocho et al., 2022), and demonstrate the effectiveness of the fish score sheet (Flueck-Giraud et al., 2025) in picking up subtle signs of welfare issues in a population.

## 11 Enrichment in aquaculture

To promote the transition from surviving to thriving in small-to-medium scale inland aquaculture, Yannick Rohrer from the Swiss fair-fish association reported about his approaches to promote natural behaviors such as shelter seeking, spatial exploration, foraging strategies, social signaling, and other inherently rewarding activities in recirculating aquaculture. Among the various categories of enrichment (structural, physical, sensory, occupational, dietary, and social (Arechavala‐Lopez et al., 2022), structural modifications are easiest to design and to implement and can also improve resilience towards other stressors; for example, enriched rainbow trout return more rapidly to baseline behavior following handling (Pounder et al., 2016).

Practical challenges for structural enrichment include dirt accumulation, impacts on tank hydraulics, reduced visibility for farmers, and potential stress for fish during cleaning procedures. For aquaculture applications, enrichment solutions must therefore be simple, robust, standardized, compatible with hydraulic requirements, easy to install and remove, and effective at commercial scale. Rohrer presented preliminary results from ongoing work evaluating a modular enrichment system in recirculating aquaculture. In the conditions tested so far, the structures showed no apparent detrimental effects and preliminary observations suggested potential improvements in feed conversion and early feeding performance. These effects will be investigated further in a larger research trial planned for 2027, where recirculating aquaculture facilities with an interest in structural enrichment are invited to participate.

## 12 What do fish think about enrichment?

One challenge in advancing positive welfare is the appropriate interpretation of conventional endpoints such as growth, weight gain, or disease susceptibility. We currently know very little about how fish truly perceive and respond to their environment, which requires a detailed understanding of their perceptions and reactions to e.g. physical structures. Rainer Friedrich from the Friedrich Miescher Institute in Basel presented how exposing zebrafish to virtual environments and measuring their movement as well as their neuronal activities reveals that zebrafish form expectations and internal representations of their surroundings (Huang et al., 2020).

Presently, such research is resource-intensive since it involves two-photon imaging of live animals under water, requires high-quality virtual environments, and encompasses extensive training of the involved researchers. However, these approaches enable targeted investigations of fishes’ perceptions of environmental structures and conspecifics, or the effect of medications on brain activity in fish, linking neuronal responses to behavioral outcomes. Providing increased accessibility in terms of infrastructure and training, these methods can be expected to become a key tool for fundamental research on positive fish welfare in the future.

## 13 Transport

In research, aquaculture, and the pet industry, fish are transported, sometimes across long distances. While the livestock industry features clear requirements regarding transport conditions, this aspect is often neglected in laboratory animal husbandry. It is currently unclear whether standard fish transport protocols (Aleström et al., 2020) are truly benign or impose hidden physiological costs that could affect the outcome of subsequent experiments.

Alexis Buatois from EAWAG in Zurich presented preliminary, unpublished results from an assessment of current best-practice transport guidelines for zebrafish in Sweden, in which adult fish were transported by car for approximately six hours and monitored for water quality, cortisol levels and behaviour over a nine-day recovery period. They reported that cortisol and behavioural indicators appeared to return towards baseline only by around day nine, and suggested, on this basis, that a recovery period of at least nine days may be warranted before commencing experimentation, to avoid stress-induced confounds. They further noted that these preliminary observations suggest current guidelines for the live transport of fish merit further consideration to improve animal welfare, for example towards including oxygenation during transport.

## 14 Implementation in research

How can high standards of fish welfare be achieved while allowing excellence in research? Joana Monteiro from the Champalimaud Foundation in Lisbon described the husbandry and management practices implemented in their centrally managed research fish facility, where trained personnel provide all the husbandry, preventive health program, and colony management, and deliver specialized research support services. This integrated approach was presented as supporting stable environmental and health conditions while reducing the time researchers spend on husbandry and experimental preparation. Monteiro described several practices adopted by the facility following internal evaluation and refinement of husbandry protocols, for example the facility’s principle that “good results depend on good materials”: Optimal husbandry and welfare lead to reliable data, improved reproducibility, and ultimately meaningful scientific progress (Ellis et al., 2012).

A key component of the facility’s operations is to approach husbandry with a scientific mindset, e.g. through continuous evaluation and refinement of their protocols based on sound data. Based on this, the facility adopted life-stage-adapted husbandry practices, with age-appropriate feeding, water quality and flow conditions. Tanks that do not receive adequate inflow are identified through infrared thermal mapping. Isolation, for example for genotyping, is carefully managed and often achieved without solitary confinement through phenotypic isolation within a group of clearly distinguishable individuals. A simple measure to preserve water quality in overnight static-water breeding tanks is to withhold the final feed before breeding, which also contributes to high spawning success and embryo survival. Health status is monitored daily through behavioral and external phenotypic observations, and complemented by regular microbiological screening and a two-stage quarantine procedure with proven success at eliminating infections. Additional health management rules include a strict no-return policy for animals leaving the facility, salt treatments for minor superficial lesions, and a defined maximum age policy.

To minimize inbreeding and its deleterious effects on fish health while maintaining valuable genetic tools, colony management relies on multiple pairwise crosses and equal offspring selection to ensure a balanced contribution of individuals to subsequent generations. Genetically modified lines are maintained by alternate incrossing and outcrossing to wild type fish. Also, regular genetic refreshment through animals from other facilities or resource centers, together with sperm cryopreservation, forms an integral part of stock management.

## 15 What’s next?

Based on these reflections, what are the most pressing needs of the fish research community regarding welfare? What is perceived as the upcoming next step in fish welfare research? During coffee and lunch breaks, participants were encouraged to propose discussion topics using sticky notes on a poster wall. These notes were then used as conversation starters during the concluding panel discussion among keynote speakers and the audience. In this discussion, the following areas emerged as needs for future development.

### 15.1 Translatability and applicability of recommendations

The discussion revealed that welfare research and resulting recommendations don’t always reflect the practical realities of research facilities, for example in terms of tank sizes or practicability in a high-throughput environment. Participants identified a need for studies conducted under conditions that resemble everyday practice, including experiments in small research tanks, at commercial aquaculture scale, across different life stages, and including burdened or mutated lines in addition to wild-type fish. Participants felt that some of the current literature on refinement and welfare in experimental fish may create unrealistic expectations among administrators and regulatory authorities. This may lead to demands for welfare measures that are difficult to implement and come with additional costs or unwanted side effects. An example named was physical enrichment, which raises concerns regarding hygiene, biosecurity, and feasibility.

### 15.2 Lifetime assessments

Participants noted that welfare assessment needs to move beyond disconnected single-point assessments towards cumulative welfare measurements across the animal’s lifetime. The first step would be to collate, analyze and publish the immense body of existing welfare-related data which are already collected by research facilities due to legal requirements. This should be accompanied by the development of improved welfare assessment tools. Several participants pointed towards the limitations of cortisol-based measures, for example the difficulty to distinguish between tank-level averages and the welfare state of individual fish (Ellis et al., 2012). It was also suggested that continuously low cortisol concentrations may not indicate good welfare and instead signify a certain unpreparedness to deal with stressors that would inevitably occur. Anecdotal evidence shared at the meeting suggested that animals which had experienced repeated low-level stressors, as opposed to no stressors, were better at recovering from stress/cortisol spikes later in life.

### 15.3 Sharing is caring

While some institutions have the technical and human resources to test and evaluate the effect of innovative housing conditions on welfare, many experimental fish facilities are small, run without a designated caretaker or veterinarian, and depend on information from other institutions for improvements. Participants therefore identified a dire need for local informal networks and called for institutionalized mechanisms to share knowledge. Such a network, or central person, would provide easy access to information, protocols, practical improvements, and the communication of unsuccessful attempts to improve welfare, e.g. failed enrichment strategies. Meeting participants considered this critical for advancing the field, for avoiding a waste of resources, for ensuring optimal welfare for the fish, and for facilitating access to vertebrate models to researchers.

### 15.4 There is more than zebrafish

Research on welfare in fish is mostly focused on zebrafish, but the experimental landscape of fish is much more diverse. Participants therefore expressed the need for a reliable legal frame and a broader taxonomic scope in directives and legislation for other species. Because zebrafish dominate the current fish husbandry literature, they are also the only species explicitly treated by law and directives with regards to species-specific needs (European Commission, 2024). Directive 2010/63/EU establishes generic care and accommodation requirements for fish used in research (European Parliament and Council of the European Union, 2010), but does not recognize that the fundamentally diverging requirements in terms of water volume, tank design, enrichment, feeding, and husbandry for this taxonomically extremely diverse species group. The conclusion was that welfare data needs to be generated across a wider range of species without compromising relevant zebrafish research, where important gaps remain. For example, surprisingly little is published about the behavior and ecology of zebrafish in their natural habitats (Lee et al., 2022). Despite their status as one of the most widely used research animals, our understanding of this model species’ wild ecology remains limited, which prevents an informed design of enrichment strategies (Lee et al., 2022).

## Data Availability

The datasets presented in this article are not readily available because they are unpublished results communicated in confidence. Requests to access the datasets should be directed to the respective mentioned presenters.
